# Evaluation of ABL90 and ABL800 Radiometer Blood Gas Analyzers: Challenges and Applications in Point-of-Care Cancer Diagnostics in Saudi Arabia

**DOI:** 10.3390/healthcare13030331

**Published:** 2025-02-06

**Authors:** Abdulaziz Yahya Al-shahrani, Johra Khan

**Affiliations:** Department of Medical Laboratory Sciences, College of Applied Medical Sciences, Majmaah University, Al Majmaah 11952, Saudi Arabia; 441104210@s.mu.edu.sa

**Keywords:** point-of-care, POCT, cancer, devices, laboratories

## Abstract

Background: Point-of-care (POC) diagnostics is an innovative approach to healthcare analysis that brings the diagnostic process closer to the patient’s immediate care setting. This study was conducted to assess POC testing devices’ use in diagnosing cancer and detecting the main challenges facing laboratory specialists. Method: A cross-sectional study was conducted on conveniently selected laboratory specialists working in the Prince Mohammed bin Abdulaziz Hospital in Riyadh for six weeks. Result: A total of 187 study participants (51% males and 49% females) were enrolled. Around one-half of them (96, 51%) were less than 30 years old, and 85% had 1–5 years of experience, with 61% (124) having no previous cancer diagnosis devices training. Most of this study’s cohort was using ABL 90 Radio meter/blood gases (45, 24%), followed by ABL 800 Radio meter/blood gases (39, 20.9%), as the main cancer diagnostic devices. Several challenges were faced by this study’s participants during their work with cancer diagnosis devices. The participants shared that some time was needed to use most of the devices, and learning how to use them was a significantly steep learning curve (2.99 ± 0.07 of participants). Most participants (113, 60.4%) carried out all the control testing, and their results were compared completely (100%) with the central laboratory. They took special precautions to keep the instruments safe (162, 86.6%). Conclusion: The correlation between type of devices used and the challenges faced during the use of POCT cancer diagnosis devices showed that there is a significant correlation between all challenges facing the participants and the type of devices (*p* = 0.001), except for the need for time to use these devices (*p* = 0.53). There are many challenges facing workers who operate point-of-care cancer diagnosis devices to a high degree.

## 1. Introduction

Cancer currently ranks as the second most prevalent cause of mortality globally, resulting in an estimated annual death toll of 7.6 million individuals. This figure represents approximately 13% of all fatalities worldwide [1]. The provision of optimal diagnostic and therapeutic strategies holds great significance in the proactive implementation of customized medicine for cancer. This methodology facilitates the categorization of cancer subtypes and permits the assessment of the most effective treatment strategy based on patient features, medical history, and observed response to therapy. The implementation of early detection methods and tailored care, often known as precision medicine, has emerged as a viable strategy to impact both the life expectancy and quality of life in individuals diagnosed with different forms of cancer [2].

In recent years, significant attention has been dedicated to the advancement of diverse methodologies aimed at the creation of effective point-of-care testing (POCT) devices for the early detection and diagnosis of cancer. The transition towards point-of-care (POC) systems represents a significant shift in diagnostic approaches away from traditional methods that rely on medical laboratories located either in or close to patient settings. POC systems offer clinicians prompt access to diagnostic information, empowering them to make more informed decisions regarding diagnosis and treatment [3]. This development has the potential to facilitate the implementation of a domestic smart healthcare monitoring system that possesses improved selectivity and sensitivity. Additionally, it can introduce robust tools for personalized medicine, encompassing the monitoring of pandemics. The primary objective of POC devices is to detect several biomarkers simultaneously and in a multiplexed manner on a single platform. This advancement aims to provide a rapid, cost-effective, and dependable quantitative analysis of biomarkers [2]. There are many POC devices used for cancer detection, including the i-STAT System, Cepheid GeneXpert, e-Nose System, Lunaphore COMET, and ABL90 and ABL800 blood gas analyzers. In Saudi Arabia, the two most commonly used POC devices are ABL90 and ABL800 blood gas analyzers. ABL90 and ABL800 blood gas analyzers are two of the most used POC devices in hospitals. However, both ABL90 and ABL800 radiometer blood gas analyzers are used for the measurement of blood gases, electrolytes, metabolites, and a number of other critical parameters; these two significantly differ in their intended use, features, and operational capabilities. The ABL90 FLEX is compact, portable, and optimized for point-of-care testing. It is designed for rapid analysis, providing results in just 35 s with a small sample volume (as low as 65 \u03bcL), making it ideal for critical care settings like emergency departments or neonatal ICUs. In contrast, the ABL800 FLEX is a larger, laboratory-based analyzer suitable for high-throughput environments. It has a wider test menu, superior quality control functions, and is more suitable for large workloads in centralized laboratories. The ABL800 also allows for automated sampling systems and higher sample volumes. While the ABL90 was designed for speed and portability mainly for bedside use, the ABL800 was designed for comprehensive testing and scalability in centralized laboratory operations [3].

Currently, the predominant practice for diagnostic disease testing involves using expensive technology in centralized or hospital-based laboratories. These facilities require the skills of highly trained experts to operate the equipment. To promote point-of-care (POC) diagnostics, optimizing and reducing the size of the tests is crucial, hence reducing the costs associated with materials, equipment, and personnel [4]. A study conducted in a major Saudi Arabian hospital found that younger healthcare providers and those with shorter employment tenure perceived more benefits and fewer barriers to HIT implementation compared to their older [5], more experienced counterparts. Additionally, female employees reported perceiving more benefits and fewer barriers than male employees [6]. Higher levels of training were associated with positive perceptions of HIT benefits and reduced perceived barriers. These findings suggest that demographic and professional factors significantly influence healthcare providers’ acceptance and utilization of HIT systems [4].

The application of “lab-on-a-chip” and biosensor technologies has facilitated the transition from conventional laboratory-based testing to portable and user-friendly alternatives that may be employed by patients or medical workers at the point-of-care. The available reports have suggested that the necessity of device portability may be mitigated if there is a timely delivery of test results. Furthermore, it is advisable to accelerate the progression of the “test-and-treat” cycle to optimize the desired outcomes for patients [5].

Furthermore, it is imperative to consider specific essential attributes, like cost-efficiency, the capacity to generate immediate results, user-friendliness, robustness, and the ability to operate without requiring the considerable preliminary processing of samples. A plausible resolution that aligns with the above criteria is the utilization of a biosensor. At its fundamental level, a biosensor utilizes a biological component, such as an antibody, enzyme, nucleic acid, lectin, or receptor, to facilitate the detection of analytes [6]. Following this, a transducer is utilized to convert the signal obtained from detection into an electrical signal, which can be objectively quantified using an appropriate readout mechanism. Therefore, biosensors hold considerable potential for detecting changes in an individual’s health status. This can be achieved by discovering anomalies in biomolecules associated with an individual’s genome, proteome, glycome, transcriptome, metabolome, or microbiome [7]. The identification of cancer sometimes requires the concurrent exploitation of many biomarkers, given the wide array of biomarkers available. The utilization of this methodology is crucial for the establishment of a robust molecular profile that can effectively indicate the existence of cancer. Glycosylation, a widely occurring and complex post-translational modification, is evident in more than 50% of proteins found in the human body. Given the extensive prevalence of glycosylation in several biological systems, it is plausible to anticipate that modifications in glycosylation may result in significantly adverse outcomes across multiple biological systems. Using point-of-care testing devices reduces the amount of time needed to diagnose several types of diseases, specifically cancer types. This study aims to enrich the literature with the most important factors affecting the successfulness of point-of-care testing devices and their challenges in diagnosing cancer, and we also aim to shed light on the main challenges faced by technicians in the Kingdom.

## 2. Materials and Methods

### 2.1. Study Design

A cross-sectional quantitative research approach was followed using self-assessed questionnaires (supplymentary file) to obtain data over six weeks from a sample of laboratory technicians and other professionals who deal with cancer diagnosis devices.

### 2.2. Study Settings

This study was conducted in Prince Mohammed bin Abdulaziz Hospital in the Riyadh region of the Kingdom of Saudi Arabia (KSA).

### 2.3. Study Population

A convenience sampling technique using the Richard Geiger equation, with a margin of error determined as 5%, a confidence level of 95%, a 50% response distribution, and a sampling size calculation, was used to obtain a final total sample size of 187 laboratory specialists.

### 2.4. Measurement Tool

This study used an anonymous online questionnaire comprising 37 items (excluding items asking for demographic data), which was developed according to two previous studies by Inaku et al. (2019) and Ye et al. (2021) [8,9]. The demographic and professional information recorded included age, gender, specialty, grade, area of work, years of experience, and attendance to any training for POC. The questionnaire was designed to evaluate (1) knowledge of operations and maintenance of POCT devices, (2) attitudes and practices about quality control testing, and (3) the possible effect of regulatory laws and challenges on the use of POCT devices in some selected hospitals.

### 2.5. Inclusion and Exclusion Criteria

Participants were eligible for inclusion in this study if they were (1) healthcare professionals and technicians working in Riyadh Hospitals in Saudi Arabia, (2) healthcare professionals and technicians of all ages and genders holding any degree, and (3) healthcare professionals and technicians with at least one year of work experience. Meanwhile, healthcare professionals and technicians who did not consent to participate in this study, those on extended leave or vacation during the study period, and those who provided incomplete or missing data were excluded.

### 2.6. Data Collection

After obtaining IRB approval from Riyadh’s second health cluster, the researcher informed all participants on the first page of the online questionnaire that this study had two parts and would be conducted in complete secrecy and with respect for their privacy. They could freely expose their identity, but their information would not be viewed. All data were treated, analyzed, and presented as group data in an Excel sheet to be analyzed using suitable statistical tests.

### 2.7. Statistical Analysis

The gathered data were examined using SPSS version 26. The analysis employed various statistical techniques and tests to evaluate the research hypotheses. The employed methodologies encompassed descriptive statistics, specifically frequencies, percentages, averages, and standard deviations, to elucidate the areas of study pursued by the participants. Moreover, ANOVA and an unpaired t-test were conducted using independent variables.

## 3. Results

A total of 187 participants in this study working in the Prince Mohammed bin Abdulaziz Hospital in the Riyadh region with diverse demographic data filled out the questionnaire. There was an approximate equality in the gender distribution (males 51%, 96), with around one-half of the cohort (96, 51%) aged less than 30 years old with several years of experience; most had 1–5 years of experience (159, 85%). Most were working in the hematology department (109, 58.3%), with the majority of participants being pathologists (91, 48.7). About 61% (124) had no prior cancer diagnosis device training. Other demographic data are represented in Table 1.

**Figure 1:**
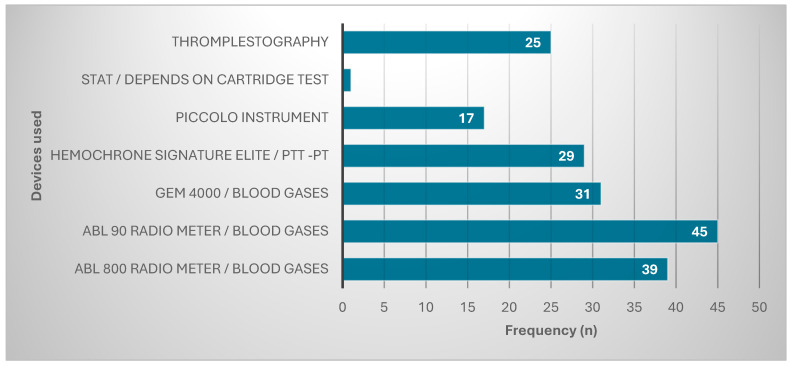
Devices used for diagnosis.

These participants used many types of devices to diagnose cancers in their hospitals. Most were using ABL 90 Radio meter/blood gases (45, 24%), followed by ABL 800 Radio meter/blood gases (39, 20.9%). The other devices used are represented in Figure 1.

The challenges facing these participants during their work with cancer diagnosis devices were several (Table 2). Per the participants, most devices need time to function (2.99 ± 0.07), and there was a large number of cancer samples (2.99 ± 0.1) coupled with a shortage of staff (2.99 ± 0.07).

Most participants agreed that devices had old technological procedures (145, 77.5%) and needed time to function (186, 99.5%). The participants disagreed that specimens were not readily prepared to be added directly (114, 61%) or that maintenance of devices was poorly performed (126, 67.4%). Other agreement and disagreement levels are represented in Table 3.

Most participants (113, 60.4%) did all control testing, and their results were compared completely (100%) with the central laboratory. They took special precautions to keep the instrument safe (162, 86.6%). Although about half of them (49.7%) were not using complete personal protective equipment (PPE), 88.8% had to wear their protective masks during work. Other precautions are represented in Table 4.

**Figure 2:**
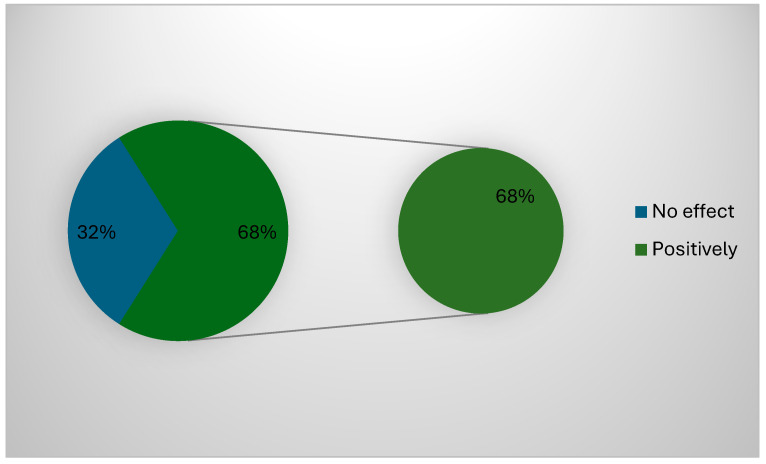
Possible effect of government regulations.

About 68% (127) of the participants confirmed that the governmental regulations positively impacted their work and applied all POCT (Figure 2). The correlation between the type of devices used and the challenges faced during the use of POCT cancer diagnosis devices showed a significant correlation between all challenges facing the participants and the type of devices (*p* = 0.001), except for the need for time to use these devices (*p* = 0.53). The correlation between demographic data used and the challenges faced during the use of POCT cancer diagnosis devices showed a significant correlation between all challenges facing the participants and area of positioning (*p* = 0.00) and years of experience (*p* = 0.02), while gender (*p* = 0.23), specialty (*p* = 0.086), or previous cancer diagnosis devices training (*p* = 0.026) showed no significant correlations (*p* > 0.05).

## 4. Discussion

This study aimed to assess point-of-care testing devices used in diagnosing cancer and detecting the main challenges facing laboratory specialists. Among 187 participants with diverse demographic data, there was an almost equal gender distribution (males 51%, n = 96), eliminating the possibility of gender bias [8]. Around half of our study’s cohort (n = 96, 51%) was less than 30 years old and had various years of experience; the majority had 1–5 years (n = 159, 85%) of experience. This aligns with several studies conducted in Saudi Arabia, which reported that most laboratory technicians in hospitals are 25–30 years old, with a maximum experience of five years [9,10,11]. Most participants worked in the hematology department (n = 109, 58.3%), the largest specialty comprising pathologists (n = 91, 48.7%), and about 61% (n = 124) had no previous cancer diagnosis device training. Setiawan et al. (2024) and Taylor et al. (2024) demonstrated how training sessions among hospital staff from different fields enhance the quality of their work [12,13].

These participants use many types of devices to diagnose cancers in hospitals. The largest proportion of them are using ABL 90 radiometer/blood gases (n = 45, 24%), followed by ABL 800 radiometer/blood gases (n = 39, 20.9%), which agrees with Malaysia et al.’s (2021) study, who found that the majority of laboratories utilize radiometer models, specifically the ABL 800 FLEX, ABL90 FLEX, and ABL80 FLEX models [14]. Yang et al. (2020) also reported that their work with the ABL90 blood gas analyzer revealed the highest accuracy when applying the POCT [15]. Also, a study by the Model of the Laboratory Medicine Relief Coordinator using POCT devices reported that the ABL 800 radiometer/blood gases is the most precise, stable, and reliable device for several cancer diagnostics.

The challenges facing the participants during their work when using cancer diagnosis devices are numerous. It has been shown that for most of the devices, there is a significant learning curve when learning to operate them (2.99 ± 0.07). Moreover, there is a large number of cancer samples (2.99 ± 0.1) and a shortage of staff (2.99± 0.7). This aligns with Mansouri et al.’s (2020) study, which found that despite the devices used in cancer diagnosis being good, the staff shortage may delay the results and affect the quality of the work [16]. Alghamdi et al. (2021) also reported that most cancer samples need more time to be diagnosed perfectly because of the large number of samples [17].

Most participants agreed that the devices have old technological procedures (n = 145, 77.5%) and that it takes time to use these devices (n = 186, 99.5%), but they disagreed that specimens are not readily prepared to be added directly (n = 114, 61%) and that the maintenance of devices is poorly performed (n = 126, 67.4%). This aligns with Al-Mandeel et al.’s (2016) study, which reported that the specimens are always well prepared and that delays in the results come from the time taken to work with the devices, as well as a study by Albadr (2019), who reported that the maintenance of devices in Saudi Arabian hospitals is perfect [18,19].

Most participants (n = 113, 60.4%) obeyed all the control testing, and their results completely matched (100%) those of the central laboratory. They also took special precautions to keep the instrument safe (n = 162, 86.6%), although about half of them (49.7%) do not use complete personal protective equipment (PPE), even though about 88.8% of them must wear protective masks during their work. This agrees with several studies reporting that wearing PPE and taking protective measurements are the main principles needed to implement the perfect POC testing among several devices and hospital work fields [20,21].

The results show a significant correlation between all challenges facing the participants and the types of POCT cancer diagnosis devices (*p* = 0.001), except for the need for time to use these devices (*p* = 0.53) (Table 5). This agrees with Lee et al.’s (2010) study, which found that the main mediating factor that makes POC easier or more difficult is the different devices used to diagnose cancer. Regarding the correlation between demographic data used and the challenges faced during the use of POCT cancer diagnosis devices, a significant correlation was observed between all challenges facing the participants and area of positioning (*p* = 0.00) and years of experience (*p* = 0.02), while gender (*p* = 0.23) and specialty (*p* = 0.086) had no significant correlation. This corresponds with Briggs et al.’s (2008) and Rempell et al.’s (2016) studies, who reported that more experience working in the hematology field enhanced the use of point-of-care testing devices among technicians [22].

## 5. Limitations

The findings of this study should be interpreted in light of several significant limitations. First, the small sample size reported in this study is the main limitation observed. Second, other healthcare professionals must be included in future studies to study other factors affecting POC, such as specimen taking and handling. Third, other devices used for diagnosing other diseases were not reported in this study.

## 6. Conclusions

Using the optimum POC precautions and regulations with the inspection of the government may enhance the work of laboratory assistants and other laboratory technicians in their use of several devices for cancer diagnosis. This study reported that many devices are continuously utilized to diagnose cancer. In the most frequently visited department (i.e., hematology), ABL 90 radiometer/blood gases and ABL 800 radiometer/blood gases are the most widely used devices. Using the appropriate PPE and obeying all the control testing regulations and staying safe during the handling and transmission of the specimen are the main POC precautions observed in this study. There are many challenges determined by the participants, such as the fact that the devices have old technological procedures and that time is needed to use these devices. They disagreed about whether specimens are not readily prepared to be added directly and about whether the maintenance of devices is poorly performed. This study underscores significant market potential for diagnostic POCT devices by revealing gaps in current technologies and practices. As cancer incidence rises globally, there is an increasing demand for rapid, accurate, and portable diagnostic solutions. Companies can capitalize on this by developing next-generation POCT devices that address the highlighted challenges, including user-friendliness, faster operational times, and modernized interfaces. Furthermore, integrating advanced features like AI-based analytics and multiplexing capabilities could cater to unmet clinical needs and expand the market.

## Figures and Tables

**Table 1:** The demographic data among the study participants (n = 187).

Demographic Data	Frequency (n)	Percentage (%)
Years of experience		
<1 year	2	1.1
>10 years	26	13.9
1–5 years	159	85.0
Area of positioning		
Cytology	8	4.3
Flow cytometry	26	13.9
Hematology	109	58.3
Histology	8	4.3
Serology	10	5.3
Specimen collection and processing	26	13.9
Specialty		
Cytotechnologist	13	7.0
Lab assistants	70	37.4
Medical technologists	13	7.0
Pathologist	91	48.7

**Table 2:** The challenges facing participants during their POCT work on cancer diagnosis devices.

Challenges of Using POCT	Mean ± S.D
Devices and chemicals used are feasible	2.59 ± 0.49
SOPs are readable	1.87 ± 0.53
There is a prior training device before practice	2.12 ± 0.67
Large number of cancer samples	2.99 ± 0.1
There is a shortage of staff	2.99 ± 0.07
Maintenance of devices is poorly performed	1.86 ± 0.55
Devices are not user-friendly	2.51 ± 0.5
Specimens are not readily prepared to be added directly	1.71 ± 0.92
Devices have old technological procedures	2.76 ± 0.47
It takes time to use devices	2.99 ± 0.07
Total	2.44 ± 0.4

**Table 3:** The level of agreement regarding the challenges of using POCT during their work on cancer diagnosis devices.

Challenges of Using POCT	Agree	Neutral	Disagree
	n (%)		
Devices and chemicals used are feasible	111(59.4)	76(40.6)	0
SOPs are readable	16(8.6)	40(21.4)	131(70.1)
There is a prior training device before practice	54(28.9)	32(17.1)	101(54)
Large number of cancer samples	185(98.9)	2(1.2)	0
There is a shortage of staff	186(99.5)	1(0.5)	0
Maintenance of devices is poorly performed	17(9.1)	44(23.5)	126(67.4)
Devices are not user-friendly	96(51.3)	0	91(48.7)
Specimens are not readily prepared to be added directly	60(32.1)	13(7)	114(61)
Devices have old technological procedures	145(77.5)	39(20.9)	3(1.6)
It takes time to use devices	186(99.5)	1(0.5)	0

**Table 4:** Participants’ main precautions and attitudes during their POCT devices’ use.

Item	Frequency (n)	Percentage (%)
Inclusion of control testing		
No	74	39.6
Yes	113	60.4
Special precautions to keep the instrument safe		
No	25	13.4
Yes	162	86.6
Results comparison with central laboratory		
Yes	187	100.0
Need For instrument validation before use		
No	107	57.2
Yes	80	42.8
Need for committee to monitor the operation of POCT devices		
No	63	33.7
Yes	124	66.3
You are making good protection from specimen		
Yes	187	100.0
You are making good protection during the transmission of the specimen		
No	44	23.5
Yes	143	76.5
Isolation is optimum for a procedure		
No	99	52.9
Yes	88	47.1
Hand hygiene		
No	8	4.3
Yes	179	95.7
Use of personal protective equipment (PPE)		
No	93	49.7
Yes	94	50.3
Use of medical protective mask		
No	21	11.2
Yes	166	88.8

**Table 5:** The correlation between type of devices used and the challenges faced during the use of POCT cancer diagnosis devices.

Challenges of Using POCT		*p*-Value
Devices and chemicals used are feasible	Types of devices used	0.000
SOPs are readable		
There is a prior training device before practice		
Large number of cancer samples		
There is a shortage of staff		
Maintenance of devices is poorly performed		
Devices are not user-friendly		
Specimens are not readily prepared to be added directly		
Devices have old technological procedures		
It takes time to use devices		0.53

## Data Availability

The data generated in this study are available in the manuscript.

## Supplementary Materials

The following supporting information can be downloaded at: https://www.mdpi.com/article/10.3390/healthcare13030331/s1, Questionnaire Form.
