# Supplementary material for: Evaluation of ABL90 and ABL800 Radiometer Blood Gas Analyzers: Challenges and Applications in Point-of-Care Cancer Diagnostics in Saudi Arabia
Source: Healthcare (Basel). 2025 Feb 6;13(3):331. doi: 10.3390/healthcare13030331 (PMC11817300; doi:10.3390/healthcare13030331)
Supplement: Supplementary file 1 [file healthcare-13-00331-s001.zip › healthcare-3363583-supplementary.pdf]

Questionnaire Form

Sociodemographic Data

**Specialty**	
Phlebotomist	Laboratory Director
Medical technologists	Histotechnologists
Pathologist	Administration
Cytotechnologist	
Lab Assistants	
**Grade**	
Junior	
Senior	
**Any previous cancer diagnosis devices training**	
No	
Yes	

1	**Age**		
	< 30 Years		
	31-40 Years		
	41- 50 years		
2	**Gender**		
	Males		
	Females		
5	**Area of work/posting**		
	Specimen collection and processing	Chemistry	Histocompatibility testing
	Cytology	Special Chemistry	Medical Microbiology
	Hematology	Toxicology	Molecular Pathology
	Serology	Immunology	Flow cytometry
	Histology	Blood Bank	

	Coagulation	Urine analysis	
6	**Total experience in years**		
	< 1 year		
	1-5 years		
	5-10 years		
	>10 years		

Instrument Validation and Quality Control Practices:

Inclusion of Control Testing

- Yes
- No
- Nil

Special Precaution to Keep the Instrument Safe

- Yes
- No
- Nil

Results Comparison with Central Laboratory

- Yes
- No
- Nil
- --

Opinion about Regulation of the use of POCT devices in the Hospital:

....

Need For Instrument Validation Before Use

- Yes
- No
- Nil

Need For Committee To Monitor The Operation of POCT Devices in Hospital

- Yes
- No
- Nil

Importance of Government Regulation

- Yes
- No
- Nil

Possible Effect of Government Regulation

- Positively
- Negatively
- No effect
- Don’t know
- Nil

Point of care practices (Yes - No - Nil)

1 Category III protection: procedure

2 Transmission of specimen

3 Isolation area

4 Category III protection: CT

5 Hand hygiene

6 Category III protection: outpatient

7 Category III protection: high risk procedure

8 Use of PPE: isolation Gown

9 Use of PPE

10 High risk procedure

1 Indicator to isolation

12 Negative pressure wards

13 Disinfection of goggles

14 Category III protection: collection sample

15 Isolation

16 Delivery of clinical specimens

17 Use of medical protective clothing

18 Use of medical protective mask

19 Use of medical protective mask

20 Use of surgical mask

21 Hand hygiene

22 Use of disposable hat

23 Use of surgical mask

24 Disinfect the environment

Point of care device barriers and challenges (Agree - Neutral - Disagree)

1. Devices and chemicals used are feasible
2. SOPs are not readable
3. No prior training devices before practice
4. Large number of cancer samples
5. There is a shortage in staff
6. Maintenance of devices is poorly performed
